# Phase II study of carboplatin/nab-paclitaxel/atezolizumab combination therapy for advanced nonsquamous non–small cell lung cancer patients with impaired renal function: RESTART trial

**DOI:** 10.1186/s12885-022-10056-x

**Published:** 2022-09-08

**Authors:** Yoshimasa Shiraishi, Junji Kishimoto, Takayuki Shimose, Yukihiro Toi, Shunichi Sugawara, Isamu Okamoto

**Affiliations:** 1 Department of Respiratory Medicine, Graduate School of Medical Sciences, Kyushu University, 3-1-1 Maidashi, Higashi-ku, Fukuoka, 812-8582 Japan; 2 Department of Research and Development of Next Generation Medicine, Kyushu University, 3-1-1 Maidashi, Higashi-ku, Fukuoka, 812-8582 Japan; 3 Department of Statistics and Data Center, Clinical Research Support Center Kyushu, 3–1-1 Maidashi, Higashi-ku, Fukuoka, 812-8582 Japan; 4 Department of Pulmonary Medicine, Sendai Kousei Hospital, 4-15 Hirose-machi, Sendai, Miyagi 980-0873 Japan

**Keywords:** Clinical trial, Nonsquamous non–small cell lung cancer, Immune checkpoint inhibitor, Chemotherapy, Renal impairment

## Abstract

**Background:**

First-line treatment of nonsquamous non–small cell lung cancer (NSCLC) has undergone a paradigm shift to platinum combination therapy together with immune checkpoint inhibitors (ICIs). However, phase III studies of combinations of cytotoxic chemotherapy and ICIs have included only patients with maintained organ function, not those with renal impairment.

**Methods:**

Cytotoxic chemotherapy–naïve advanced nonsquamous NSCLC patients aged 20 years or older with impaired renal function (creatinine clearance of 15 to 45 mL/min) are prospectively registered in this single-arm phase II study and receive combination therapy with carboplatin, nanoparticle albumin-bound (nab-) paclitaxel, and atezolizumab. Individuals with known genetic driver alterations including those affecting *EGFR*, *ALK*, *ROS1*, *BRAF*, *MET*, *RET*, and *NTRK* are excluded. We plan to enroll 40 patients over 2 years at 32 oncology facilities in Japan. The primary end point is confirmed objective response rate.

**Discussion:**

If the study demonstrates efficacy and safety of carboplatin/nab-paclitaxel/atezolizumab, then this combination regimen may become a treatment option even for nonsquamous NSCLC patients with impaired renal function.

**Trial registration:**

Registered with Japan Registry for Clinical Trials on 25 February 2021 (jRCTs071200102).

## Background

The introduction of immune checkpoint inhibitors (ICIs) that target programmed cell death–1 (PD-1), its ligand PD-L1, or cytotoxic T lymphocyte–associated protein–4 (CTLA-4) has markedly improved the survival of patients with non–small cell lung cancer (NSCLC). Phase III studies of chemotherapy-naïve patients with advanced nonsquamous NSCLC have revealed that combinations of platinum-based chemotherapy and ICIs confer longer survival compared with platinum-based therapy alone, with these findings having established such combinations as new standard treatments [1–5]. However, these phase III studies included only individuals with maintained general organ function, having excluded those with renal impairment.

Pemetrexed is the most commonly used cytotoxic drug for the treatment of advanced nonsquamous NSCLC because of its efficacy, convenient administration, and high feasibility. Pemetrexed-platinum regimens are therefore frequently adopted as a platform for the development of chemotherapy-immunotherapy combinations [1, 3, 5]. However, limited data are available for pemetrexed administration in patients with a creatinine clearance (CCr) of < 45 mL/min, and the drug label information states that pemetrexed should be withheld if CCr is < 45 mL/min. Clinical trials that have evaluated pemetrexed-containing regimens to date have stipulated a CCr of ≥45 mL/min as an eligibility criterion [1, 3, 5–7]. Even in daily clinical practice, pemetrexed-platinum regimens with or without ICIs are avoided for advanced nonsquamous NSCLC patients with a CCr of < 45 mL/min.

The combination of carboplatin and nab-paclitaxel (CnP) with the anti–PD-L1 antibody atezolizumab is also a chemotherapy-ICI combination established for nonsquamous NSCLC. In the IMpower130 trial, CnP with atezolizumab conferred prolonged survival compared with CnP alone [2]. Nab-paclitaxel is an albumin-bound nanoparticle formulation of paclitaxel, which is metabolized in the liver [8], and the pharmacokinetics of nab-paclitaxel have been found to be similar regardless of renal function [9]. Subgroup analysis of the CA031 study, a pivotal phase III trial of CnP, revealed that this regimen was as effective and safe in patients with impaired renal function (CCr of ≤50 mL/min) as in those with normal renal function [10]. In addition, an integrated analysis of clinical trials that evaluated CnP (ABOUND.SQM, ABOUND.PS2, ABOUND.70+, and CA031) indicated that it is broadly applicable not only to the general NSCLC population but also to various subgroups of patients such as those with moderate renal impairment (estimated glomerular filtration rate [eGFR] of 30 to 60 mL min^− 1^ 1.73 m^− 2^) [11]. Although the safety of CnP has not been assessed for patients with severe renal impairment (eGFR of ≤30 mL min^− 1^ 1.73 m^− 2^), given that nab-paclitaxel is metabolized in the liver and that carboplatin dose is adjusted according to renal function, we hypothesized that CnP may be safe and effective in such patients.

The frequency of toxicities of grade 3 or 4 associated with atezolizumab treatment was found to be slightly higher in advanced NSCLC patients with renal impairment than in those with maintained renal function, but the efficacy and frequencies of treatment-related death and of toxicities necessitating treatment discontinuation were similar in both populations [12]. A subgroup analysis of the expanded access program for patients with metastatic urothelial carcinoma also showed that the efficacy and safety of atezolizumab were similar regardless of the level of renal function [13].

Given this background, CnP with atezolizumab is a promising treatment option even for advanced nonsquamous NSCLC patients with impaired renal function, for whom access to pemetrexed-platinum-ICI combination regimens has been difficult. However, the efficacy and safety of CnP-atezolizumab have not been prospectively evaluated in patients with renal insufficiency. We have now designed a single-arm phase II study to evaluate the efficacy and safety of the combination of CnP with atezolizumab for patients with advanced nonsquamous NSCLC and impaired renal function.

## Methods

### Study design

**Fig. 1:**
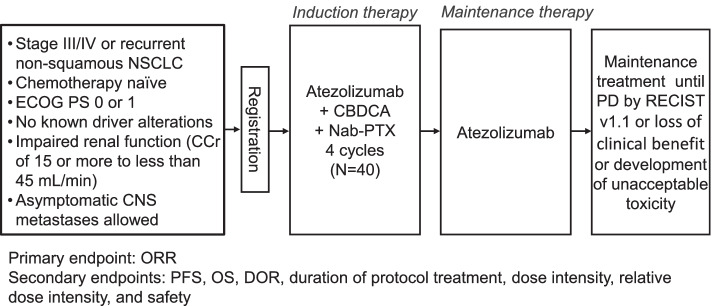
Design of the RESTART trial. NSCLC, non–small cell lung cancer; ECOG PS, Eastern Cooperative Oncology Group performance status; CCr, creatinine clearance; CNS, central nervous system; CBDCA, carboplatin; nab-PTX, nanoparticle albumin-bound paclitaxel; PD, progressive disease; RECIST v1.1, Response Evaluation Criteria in Solid Tumors version 1.1; ORR, objective response rate; PFS, progression-free survival; OS, overall survival; DOR, duration of response

The trial, RESTART (LOGIK2002), is a multicenter, single-arm phase II study designed to investigate the efficacy and safety of carboplatin/nab-paclitaxel/atezolizumab combination therapy for individuals with advanced nonsquamous NSCLC and impaired renal function (Fig. 1). Eligible patients are registered prospectively.

### Treatment plan

Atezolizumab is administered at 1200 mg on day 1 of consecutive 3-week cycles. Carboplatin is administered at an initial dose determined by Calvert’s formula to yield an area under the concentration-time curve of 5 mg mL^− 1^ min on day 1 of each 3-week cycle [14]. Nab-paclitaxel is administered at a dose of 100 mg/m^2^ on days 1, 8, and 15 of each 3-week cycle. After four cycles of induction therapy, maintenance therapy with atezolizumab is administered until disease progression, loss of clinical benefit, or development of unacceptable toxicity.

### Eligibility criteria

Individuals 20 years of age or older with histologically or cytologically confirmed nonsquamous NSCLC and impaired renal function (CCr of ≥15 but < 45 mL/min as calculated by the method of Cockcroft and Gault [15]) are eligible. (We decided to exclude patients with a CCr of < 15 mL/min because, in cases of chronic kidney disease, the introduction of maintenance hemodialysis is generally considered when the eGFR falls to ≤15 mL min^− 1^ 1.73 m^− 2^.) Patients with sensitizing driver genetic alterations of *EGFR*, *ALK*, *ROS1*, *BRAF*, *MET*, *RET*, or *NTRK* are ineligible. Each patient is required to be at clinical stage III without indication for definitive thoracic radiotherapy, to be at stage IV, or to have recurrent disease after surgery or definitive radiotherapy that is not curable by local therapy. Patients must not have been treated previously with either cytotoxic chemotherapy or ICIs. The expression level for PD-L1 on tumor cells is not a determinant of eligibility. An Eastern Cooperative Oncology Group performance status of 0 or 1 as well as adequate lung, bone marrow, and liver function are required. Asymptomatic central nervous system metastases are permitted.

Patients are not eligible for the study if they have synchronous double or multiple cancers or have had metachronous double or multiple cancers within 2 years; have active hepatitis B or active hepatitis C or other infectious disease requiring systemic treatment; show obvious interstitial lung disease on chest computed tomography (CT); are receiving continuous systemic corticosteroid or immunosuppressant treatment; have other serious medical conditions including uncontrolled diabetes, unstable angina, and clinically serious arrhythmia; manifest peripheral neuropathy of grade ≥ 2; have hypersensitivity to carboplatin, nab-paclitaxel, or atezolizumab or to formulation additives; are affected by a psychological disorder that makes it difficult to participate in the study; or are pregnant, within 28 days after parturition, or breast feeding.

### Evaluation of response and safety

A CT or magnetic resonance imaging scan of the brain, CT scans of the chest and abdomen, and a bone scan or positron emission tomography scan are required before onset of the study treatment. Contrast agents are not needed for the imaging tests. Patients undergo tumor assessment at baseline, every 6 weeks during the first 24 weeks, every 9 weeks for the next 27 weeks, and every 12 weeks thereafter. Tumor response is evaluated in accordance with the Response Evaluation Criteria in Solid Tumors (RECIST, version 1.1). Adverse events are recorded based on the National Cancer Institute Common Terminology Criteria for Adverse Events (version 5.0).

### Study endpoints

The primary end point is confirmed objective response rate, with secondary end points including progression-free survival, overall survival, duration of response, duration of protocol treatment, dose intensity, relative dose intensity, and safety. Subgroup analysis is stipulated for patients with a CCr of 15 to 30 mL/min or 30 to 45 mL/min, and the safety of CnP-atezolizumab will be evaluated with close monitoring for patients with a CCr of 15 to 30 mL/min.

### Statistical considerations

The primary end point is confirmed objective response rate. The expected response rate of this regimen in advanced nonsquamous NSCLC patients with impaired renal function is assumed to be 49.2%, which corresponds to that for the CnP-atezolizumab group in the IMpower130 study [2], and the threshold response rate is assumed to be 31.9%, which corresponds to that for the CnP group in the same study. The necessary sample size is estimated to be 35 subjects, with a one-sided significance level (α value) of 10% and a power of 80%. Taking into account ineligible subjects and those lost to follow-up, the target sample size was determined to be 40 subjects.

## Discussion

As far as we are aware, this is the first prospective study to evaluate the combination of cytotoxic chemotherapy with an ICI for nonsquamous NSCLC patients with impaired renal function. If the study demonstrates efficacy and safety for CnP with atezolizumab in this patient population, then this regimen will become a potential treatment option for such individuals in daily clinical practice. This trial is based at 32 oncology centers in Japan. Patient enrollment was initiated in March 2021 and is to continue for 2 years.

## Data Availability

Not applicable.

## Abbreviations

ICI: Immune checkpoint inhibitor
PD-L1: Programmed cell death–ligand 1
NSCLC: Non–small cell lung cancer
CCr: Creatinine clearance
CnP: Carboplatin and nab-paclitaxel
eGFR: Estimated glomerular filtration rate
CT: Computed tomography
